# Association of serum inflammatory factors and hematologic parameters with lower limb deep vein thrombosis in neurocritical patients: A retrospective cohort study

**DOI:** 10.5937/jomb0-58954

**Published:** 2026-03-17

**Authors:** Yunqiu Zhu, Chun Wang, Jia Wang, Tian Shi, Weijie Tang, Huaping Zhou, Wanfei Guo, Hongbin Mei, Jixiang Bo

**Affiliations:** 1 Yangzhou University, School of Nursing and School of Public Health, Yangzhou, China; 2 Northern Jiangsu People's Hospital, Department of Neurointensive Care Unit, Yangzhou, China; 3 Northern Jiangsu People's Hospital, Department of Cardiovascular Medicine, Yangzhou, China

**Keywords:** neurocritical care, Deep Vein Thrombosis, coagulation-inflammation interaction, D-dimer, neurokritična nega, duboka venska tromboza, interakcija koagulacije i upale, D-dimer

## Abstract

**Background:**

The aim of this study was to investigate the mechanisms of postoperative deep vein thrombosis (DVT) of the lower extremities in neurocritical care patients, focusing on the interaction of inflammatory factors [D-dimer (DD), prothrombin time (PT), blood cell parameters (lymphocytes, platelets, plateletcrit) and coagulation function.

**Methods:**

A retrospective cohort study design was used to include 261 neurocritical care patients who underwent surgery between August 2019 and August 2021. They were categorized into DVT group (n = 121) and non-DVT group (n = 140) based on postoperative lower limb Doppler ultrasound findings. Preoperative coagulation indices (TT, DD), inflammatory markers [C reactive protein (CRP), procalcitonin (PCT), fibrinogen (FIB)], and blood cell parameters (hemoglobin, neutrophils, lymphocytes, platelet counts, and plateletcrit) were collected from the patients.

**Results:**

DD levels were significantly higher and TT shorter in the DVT group than in the non-DVT group (P&lt; 0.05); DD was positively correlated with PT-INR (P&lt; 0.001). In addition, lymphocyte and plateletcrit were significantly lower in the DVT group (P&lt; 0.05). Multifactorial analysis showed that age (OR = 1.027), days of hemostatic medication (OR = 1.203), postoperative infections (OR= 2.728), elevated DD (OR = 1.056), and shortened TT (OR = 0.840) were independent risk factors (P&lt; 0.05).

**Conclusions:**

The development of DVT in neurocritical patients is the result of a combination of hypercoagulation, inflammatory response, and immunosuppression.

## Abstract (sr)

**Uvod:**

Cilj ove studije bio je da se istraže mehanizmi postoperativne duboke venske tromboze (DVT) donjih ekstremiteta kod pacijenata na neurokritičnoj nezi, fokusirajući se na interakciju inflamatornih faktora [D-dimer (DD), protrombinsko vreme (PV), parametri krvnih zrnaca (limfociti, trombociti, trombocitkrit) i koagulaciona funkcija.

**Metode:**

Korišćen je retrospektivni kohortni dizajn studije kako bi se obuhvatilo 261 pacijent na neurokritičnoj nezi koji su podvrgnuti operaciji između avgusta 2019. i avgusta 2021. godine. Pacijenti su kategorisani u grupu sa dubokom venskom trombozom (n = 121) i grupu bez duboke venske tromboze (n = 140) na osnovu postoperativnih nalaza dopler ultrazvuka donjih ekstremiteta. Od pacijenata su prikupljeni preoperativni indeksi koagulacije (TT, DD), inflamatorni markeri [C-reaktivni protein (CRP), prokalcitonin (PCT), fibrinogen (FIB)] i parametri krvnih zrnaca (hemoglobin, neutrofili, limfociti, broj trombocita i trombocitkrit.

**Rezultati:**

Nivoi DD (diferencijalne preferencije) bili su značajno viši, a TT (trajni osećaj trajanja) kraći u grupi sa DVT nego u grupi bez DVT (P&lt; 0,05); DD je bio pozitivno koreliran sa PT-INR (P&lt; 0,001). Pored toga, limfociti i trombociti su bili značajno niži u grupi sa DVT (P&lt; 0,05). Multifaktorska analiza je pokazala da su starost (OR= 1,027), dani hemostatske terapije (OR = 1,203), postoperativne infekcije (OR= 2,728), povišen DD (OR= 1,056) i skraćeni TT (OR= 0,840) nezavisni faktori rizika (P&lt; 0,05).

**Zaključak:**

Razvoj duboke venske tromboze kod neurokritičnih pacijenata je rezultat kombinacije hiperkoagulacije, inflamatornog odgovora i imunosupresije.

## Introduction

Deep vein thrombosis (DVT) is a venous reflux disorder caused by abnormal coagulation of blood in the deep veins [1]. DVT is more common in patients after major surgery or severe trauma. The main causes are venous wall damage, slow blood flow, and hypercoagulable state. At present, clinically confirmed risk factors that may be involved in the formation of DVT include surgery, immobilization, tumors, family history and other factors. Statistics show that the incidence of DVT in ICU patients can be as high as 80% [2]. Due to the unique nature of neurocritical illness, patients often present with consciousness disorders, paralysis, and other symptoms that prevent timely identification of early clinical signs. These patients are unable to cooperate with early diagnostic tests such as the Homans' sign and Neuhof's sign, making them a high-risk group for DVT [3]. The NCS evidence-based guidelines point out that the incidence of postoperative DVT in neurosurgery patients ranges from 0% to 15.5%, which is a high incidence [4]. Although a certain consensus has been formed for the prevention and diagnosis of DVT [5], the autonomic dysfunction, inflammatory storm, and imbalance of the coagulation-anticoagulation system in neurocritical care patients due to central nervous system injury make the mechanism of DVT more complex, and more in-depth pathophysiological exploration is urgently needed.

In recent years, the interaction between inflammatory response and coagulation function has become a hot spot in the study of thrombotic diseases [6]. Inflammatory factors not only serve as markers of the degree of inflammation, but may also be directly involved in the process of thrombosis by mediating endothelial dysfunction, platelet activation, and the coagulation cascade [7]. For example, CRP promotes thrombin generation through upregulation of tissue factor expression [8]; while elevated levels of FIB, a key protein for coagulation, may reflect both hypercoagulable state and active inflammation [9]. In addition, dynamic changes in blood cell parameters (e.g., neutrophil/lymphocyte ratio, platelet count, hemoglobin concentration, etc.) are not only a direct manifestation of the body's inflammatory response, but may also indirectly regulate the risk of thrombosis by affecting the rheological properties of the blood and the vascular endothelial microenvironment [10].

In this study, we will focus on patients with neurocritical illness combined with lower limb DVT and systematically analyze the expression characteristics of serum inflammatory factors [C-reactive protein (CRP), D-dimer (DD), procalcitonin (PCT), fibrinogen (FIB)] and blood cell parameters (hemoglobin, neutrophils, lymphocytes, platelets, hematocrit), and further explore them with the coagulation function indicators. This study is the first to integrate the multidimensional data of inflammatory factor profiles, blood cell dynamics and coagulation function, revealing the interaction network of the three in neurocritical DVT, providing theoretical basis for the development of prediction models based on the joint analysis of multiple indexes and individualized anticoagulation strategies, and laying the foundation for the in-depth analysis of the mechanism of the neuroimmunocoagulation axis in thrombosis.

## Materials and methods

### General information

A retrospective study was conducted, selecting clinical data from 261 neurocritically ill patients who required surgical treatment and were admitted to the hospital between August 2019 and August 2021. Sample size was calculated based on a previous study reporting a DVT incidence of 15.4% in neurosurgical patients [11], with a power of 80% and α=0.05, requiring a minimum of 242 patients. Based on the results of Doppler ultrasound examination of the lower limbs performed when patients were admitted to the intensive care unit (ICU) post-surgery, they were divided into two groups: DVT group (n=121) and non-DVT group (n=140) (postoperative lower limb Doppler ultrasound was performed within 72 hours of ICU admission and weekly thereafter until discharge or DVT detection).

### Inclusion criteria

Inclusion criteria: Neurological diseases,including traumatic brain injury, cerebrovascular diseases, etc.; Age 18 years; Hospitalization within 24 hours of onset; ICU stay 3 days; No history of DVT; Ultrasound examination of limb veins within 24 hours of onset showed negative venous disease; Complete clinical data. Exclusion criteria: Patients with special treatment during hospitalization, such as radiotherapy, chemotherapy, and hemofiltration dialysis; Combined blood system diseases (e.g., leukemia, thrombocytopenia, coagulation disorders); Combined severe liver or kidney diseases; Combined malignant tumors; Vascular diseases such as lower extremity venous thrombosis or venous sinus thrombosis before admission.

### DVT Diagnose Criteria and Grouping

All neurocritically ill patients were diagnosed according to the diagnostic criteria in *Ultrasound for Lower Extremity Deep Venous Thrombosis: Multidisciplinary Recommendations From the Society of Radiologists in Ultrasound Consensus Conference* [12] after surgery. The lower limbs were examined regularly every week using a portable color ultrasound diagnostic system SII (FUJIFILM SonoSite, Inc., Device Registration No. 20182060347) until DVT was detected or the patient recovered and was discharged from the hospital.

### Inflammatory and coagulation indicators testing methods

Preoperative fasting venous blood (5 mL) was collected from patients. Serum was separated by centrifugation using a centrifugal blood component separator (AmiCORE, Fresenius Kabi AG, Device Registration No. 20183100418) at 3200 r/min for 10 minutes with a radius of 12 cm. Serum levels of CRP DD, PCT, and fibrinogen (FIB) were measured using a fully automatic biochemical analyzer (XT 3400, Ortho-Clinical Diagnostics, Inc., Device Registration No. 20222220393).

### Blood cell detection method

Fasting venous blood (5 mL) was collected 24h before surgery. Hemoglobin, neutrophil, and lymphocyte levels were analyzed using a flow cytometer BD FACSLyric (BD Biosciences, Device Registration No. 20192220383). The platelet function analyzer VerifyNow (Accriva Diagnostics, Inc., Device Registration No. 20242220383) was used to detect the patient's platelet count and plateletcrit.

### Coagulation function testing method

Preoperative fasting venous blood (5 mL) was collected. The prothrombin time (PT), thrombin time (TT), and activated partial thromboplastin time (APTT) were detected using a fully automatic coagulation analyzer CP3000 (SEKISUI MEDICAL CO., LTD., Device Registration No. 20182220294), and the PT international normalized ratio (PT-INR) was calculated.

### Statistical methods

Data processing was performed using SPSS version 25.0. *Shapiro-Wilk* test was used to verify the normality of the measurement data. The data that met the normal distribution were expressed as » χ±s«, and the independent sample t test was used for comparison between the two groups. The data that did not meet the normal distribution were expressed as »M [*P25, P75*]«. The nonparametric *Mann-Whitney U* test was used for comparison between the two groups. The count data were expressed as n% and the chi-square test was used. Spearman was used to analyze the relationship between each factor and the coagulation function of neurocritically ill patients. Statistical significance was set at α=0.05.

## Results

### Comparison of baseline data between the DVT group and the Non-DVT group

Gender, age, body mass index, bed rest time, the number of catheterizations, dehydration frequency, days of anticoagulant use, days of hemostatic use, intraoperative blood transfusion, postoperative drainage tube, postoperative infection, underlying diseases, and lifestyle habits were analyzed between the two groups. The age, number of catheter insertions, and days of hemostatic medication were higher in the DVT group than in the Non-DVT group, and the proportion of patients with postoperative infections was higher than in the Non-DVT group (*P* < 0.05, Table 1).

**Table 1:** Comparison of baseline data between DVT group and non-DVT group.

Data		DVT group (n=121)	Non-DVT group (n=140)	χ^2^/Z	P
Gender	Male	69 (57.02)	80 (57.14)	0.000	0.985
	Female	52 (42.98)	60 (42.86)		
Age (years)		[65.00 (55.00, 71.00)]	[57.00 (50.50, 67.50)]	2.775	0.006
Body Mass Index (kg/m^2^)		[23.56 (22.03, 26.13)]	[24.80 (21.98, 27.12)]	1.557	0.120
Bed Rest Time (d)		[13.00 (9.00, 19.00)]	[14.00 (9.00, 22.00)]	1.542	0.123
The Number of Catheterizations (times)		[1.00(0.00, 1.00)]	[1.00(0.00, 1.00)]	2.039	0.041
Dehydration Frequency (times/day)		[4.00 (3.00, 4.00)]	[3.00 (3.00, 5.00)]	0.250	0.802
Days of Anticoagulant Use (d)		[0.00(0.00, 1.00)]	[0.00(0.00, 4.00)]	1.177	0.239
Days of Hemostatic Drugs Use (d)		[2.00 (1.00, 3.00)]	[2.00 (1.00, 3.00)]	2.137	0.033
Blood Transfusion During Surgery	Yes	17 (14.05)	10 (7.14)	3.338	0.068
	No	104 (85.95)	130 (92.86)		
Postoperative Drainage Tube	Yes	78 (64.46)	88 (62.86)	0.072	0.788
	No	43 (35.54)	52 (37.14)		
Postoperative Infection	Yes	104 (85.95)	91 (65.00)	15.079	<0.001
	No	17 (14.05)	49 (35.00)		
Underlying Disease	Hypertension	68 (56.20)	82 (58.57)	0.150	0.699
	Diabetes	12 (9.92)	22 (15.71)	1.925	0.165
	Hyperlipidemia	2 (1.65)	3 (2.14)	0.083	0.773
Lifestyle	Smoking	32 (26.45)	44 (31.43)	0.781	0.377
	Drinking	32 (26.45)	46 (32.86)	1.273	0.259

### Comparison of preoperative coagulation function between the DVT group and the Non-DVT group

The results in Table 2 showed that the preoperative TT in the DVT group was shorter than that in the non-DVT group (*P* < 0.05). However, there was no statistical difference in PT, APTT, and PT-INR between the two groups (*P* > 0.05).

**Table 2:** Comparison of preoperative coagulation function between DVT group and non-DVT group.

Group	PT	TT	APTT	PT-INR
DVT group (n=121)	[13.20 (11.90, 14.20)]	[16.70 (15.80, 17.60)]	[28.80 (25.90, 36.40)]	[1.09 (1.04, 1.19)]
Non-DVT group (n=140)	[13.10 (12.20, 14.00)]	[17.15 (15.75, 18.45)]	[30.85 (26.95, 36.35)]	[1.08 (1.03, 1.16)]
Z	0.082	2.072	1.251	0.812
P	0.934	0.038	0.211	0.417

### Comparison of preoperative inflammation indicators between the DVT group and the Non-DVT group

The results in Table 3 showed that the preoperative DD in the DVT group was higher than that in the non-DVT group ([4.52 (1.49, 10.46)] mg/L) (*P* < 0.05). However, there was no statistical difference in CRP PCT, and FIB between the two groups (*P* > 0.05).

**Table 3:** Comparison of preoperative inflammation and coagulation indicators between DVT group and non-DVT group.

Group	CRP (mg/L)	DD (mg/L)	PCT (ng/L)	FIB (g/L)
DVT group (n=121)	[58.15 (19.30, 110.53)]	[4.52 (1.49, 10.46)]	[0.18 (0.06, 0.59)]	[3.31 (2.60, 4.41)]
Non-DVT group (n=140)	[48.00 (10.96, 92.80)]	[2.21 (1.03, 4.87)]	[0.13 (0.50, 0.30)]	[3.10 (2.42, 4.80)]
Z	0.889	3.991	1.957	0.335
P	0.374	<0.001	0.050	0.737

### Comparison of blood cell counts before operation between DVT group and Non-DVT group

The results in Table 4 showed that the hemoglobin, lymphocyte, and plateletcrit in the DVT group [109.50 (93.50, 128.50)] g/L, [0.78 (0.56, 1.03)]x10^9^/L, [0.18 (0.14, 0.22)] % were lower than those in the non-DVT group [120.50 (104.00, 137.00)] g/L, [0.90 (0.64, 1.33)]x10^9^/L, [0.20 (0.16, 0.24)] % (*P*<0.05).

**Table 4:** Comparison of blood cell count before operation between DVT group and non-DVT group.

Group	Hemoglobin (g/L)	Neutrophils (x10^9^/L)	Lymphocytes (x10^9^/L)	Platelets (x10^9^/L)	Plateletcrit (%)
DVT group (n=121)	[109.50 (93.50, 128.50)]	[10.89 (8.20, 13.93)]	[0.78 (0.56, 1.03)]	[150.50 (116.50, 193.50)]	[0.18 (0.14, 0.22)]
Non-DVT group (n=140)	[120.50 (104.00, 137.00)]	[10.28 (7.39, 12.70)]	[0.90 (0.64, 1.33)]	[166.00 (129.50, 204.50)]	[0.20 (0.16, 0.24)]
Z	2.820	1.235	2.453	1.880	2.153
P	0.005	0.217	0.014	0.060	0.031

### Analysis of correlates affecting DVT

Variables with P<0.05 in univariate analysis were entered into a backward stepwise logistic regression model. Collinearity was assessed using variance inflation factor (VIF). Finally, we performed a logistic regression analysis of the relevant factors affecting DVT, and the results showed that Age (OR=1.027), Days of Hemostatic Drug Use (OR=1.203), Postoperative Infection (OR=2.728), DD (OR=1.056), and TT (OR=0.840) were all independent factors affecting DVT (*P* < 0.05, Table 5). The Hosmer-Lemeshow goodness-of-fit test showed adequate model calibration (χ^2^=8.23, P=0.41).

**Table 5:** Multifactorial analysis of factors affecting DVT

Influencing factors	* B *	* SE *	* Wald *	* P *	* OR *	95% Confidence
Age	0.027	0.013	4.500	0.034	1.027	1.002~1.052
Number of Catheterizations	0.290	0.209	1.937	0.164	1.337	0.888~2.013
Days of Hemostatic Drug Use	0.185	0.093	3.971	0.046	1.203	1.003~1.442
Postoperative Infection	1.003	0.362	7.699	0.006	2.728	1.343~5.541
DD	0.055	0.019	8.124	0.004	1.056	1.017~1.097
Hemoglobin	-0.004	0.007	0.335	0.563	0.996	0.982~1.010
Lymphocytes	-0.471	0.260	3.278	0.070	0.624	0.375~1.040
Plateletcrit	-0.715	2.186	0.107	0.743	0.489	0.007~35.469
TT	-0.174	0.078	4.918	0.027	0.840	0.721~0.980
Constant	0.586	1.588	0.136	0.712	-	-

## Discussion

Due to paralysis or prolonged coma, neurocritically ill patients suffer from venous blood stasis, which increases the formation, expansion and consolidation of blood clots. Additionally, cerebrovascular diseases, such as ischemic stroke and hemorrhagic stroke, can also increase the risk of postoperative DVT by affecting vascular endothelial function [13][14]. Surgery for neurocritically ill patients is often more complicated. In one study, ultrasound examination of 233 patients 3 to 10 days after major surgery revealed that the incidence of DVT was 47.64%, of which the incidence in general surgery was 49.29%, 47.21% in urology, 35% in orthopedics, and as high as 53.95% in neurosurgery [15]. It can be seen that neurocritically ill patients have a higher risk of postoperative DVT This study analyzed the clinical characteristics and laboratory indicators of neurocritically ill patients and found that age, number of days of hemostatic drug use, high levels of DD and TT, and postoperative infection were risk factors for postoperative DVT in neurocritically ill patients, and DD was the most important influencing factor.

First, we observed that DD was significantly elevated and TT was decreased in the DVT group, a result that reveals a central driver of coagulation-inflammation interaction. As a fibrin degradation product, I hypothesized that DD might be involved in DVT formation through the following mechanisms: (1) Inflammation-mediated hypercoagulability: the elevation of DD is closely related to postoperative infections [16], which can induce systemic inflammatory responses, upregulate tissue factor expression, activate exogenous coagulation pathways, and promote thrombin generation [17]. (2) Endothelial dysfunction: DD exacerbates thrombosis by binding to receptors on the surface of vascular endothelial cells, inducing endothelial cell apoptosis and procoagulant phenotypic transformation [18]. In addition, TT shortening in the DVT group directly reflects enhanced thrombin activity, accelerating the conversion of fibrinogen to fibrin and the formation of a stable thrombus network [19]. Although fibrinogen levels were similar between groups, TT shortening may reflect increased thrombin activity rather than fibrinogen quantity, potentially due to dysregulated fibrinolysis or platelet hyperreactivity. This finding suggests that the coagulation system of neurocritical care patients is »hyperresponsive« in the postoperative period, which may be related to the autonomic dysfunction and inflammatory factor release caused by central nervous system injury.

As for blood cells, we observed a significant decrease in lymphocytes in the DVT group, and the mechanism may involve imbalance of the immune-coagulation axis: lymphopenia may weaken anti-inflammatory and anticoagulant regulation (e.g., reduce protein C pathway activation), exacerbate endothelial damage and platelet activation, and form a pro-thrombotic microenvironment [20]. In addition, although there was no difference in platelet counts between the DVT and Non-DVT groups, plateletcrit was reduced in the DVT group, which suggests that platelets were heavily recruited to the thrombus site during our thrombosis, resulting in a decrease in circulating platelet number and pressure volume. Meanwhile, the hyperinflammatory state of neurocritical patients may induce enhanced platelet activation (e.g., increased expression of P-selectin), which promotes platelet aggregation and procoagulant particle release, accelerating thrombus progression [21].

Finally, by logistic regression analysis, we observed that Age, days of hemostatic drug use, post-operative Infection, DD, and TT were independent factors affecting DVT, and these findings suggest that DD and TT will probably be new clinical indicators for DVT risk assessment. Subsequently, we can be alerted to the risk of DVT based on their expression thresholds by routinely testing DD and TT levels after surgery. In patients with high DD or short TT, direct oral anticoagulants (e.g., rivaroxaban) are preferred to inhibit thrombin activity and reduce fibrin generation. However, the results of lymphocyte and plateletcrit analyses showed no association with DVT, suggesting that they are not independent factors affecting DVT, but this may also be due to the high number of confounding factors in the retrospective analyses, and further validation analyses are to follow. Based on the results of this article, we believe that in the future, the clinical assessment of immune status can also be done through the combined lymphocyte counts, and the lowering of lymphocytes suggests the need to strengthen infection prevention and control and immunomodulation. In addition, dynamically adjusting the use of antiplatelet drugs (e.g., aspirin) according to plateletcrit may also help to avoid the risk of bleeding due to excessive platelet depletion.

This study also has its limitations. This study is a single-center retrospective study, and there may be selection bias in the process of patient inclusion. Furthermore, due to the limitations of clinical data, some possible related factors were not included in the study. In the future, a prospective study can be conducted through joint multicenters to discover more factors that may affect the occurrence of postoperative DVT in neurocritically ill patients.

## Conclusion

Our findings highlight the central role of hypercoagulability (elevated DD, shortened TT) and inflammation (postoperative infection) in DVT pathogenesis. While lymphopenia showed a trend toward association, further prospective studies are needed to validate its clinical utility.

## Dodatak

### Availability of data and materials

The data used to support the findings of this study are available from the corresponding author upon request.

### Funding

This research was supported by Northern Jiangsu. People's Hospital Scientific Research Fund Project (Project Number: SBKY21074).

### Conflict of interest statement

All the authors declare that they have no conflict of interest in this work.
